# Reconceptualizing Intrauterine Resuscitation and Its Short-Term Impact

**DOI:** 10.3390/diagnostics15030255

**Published:** 2025-01-23

**Authors:** Lawrence D. Devoe, David W. Britt, Christian R. Macedonia, Jaqueline M. Worth, George M. Mussalli, Myriam Mondestin-Sorrentino, Mark I. Evans

**Affiliations:** 1 Department of Obstetrics and Gynecology, Medical College of Georgia at Augusta University, Augusta, GA 30912, USA; ldevoe@augusta.edu; 2 Fetal Medicine Foundation of America, New York, NY 10128, USA; dwbrit01@me.com; 3 Lancaster Maternal Fetal Medicine, Lancaster PA and School of Pharmaceutical Sciences College of Pharmacy, University of Michigan, Ann Arbor, MI 48109, USA; christian.macedonia@gmail.com; 4 Department of Obstetrics, Gynecology, and Reproductive Sciences, Icahn School of Medicine at Mount Sinai, New York, NY 10029, USA; drw@villiageob.com (J.M.W.); drm@villageob.com (G.M.M.); 5 Princeton Perinatal Institute, Lawrenceville, GA 30046, USA; mondesmy@gmail.com; 6 Department of Obstetrics and Gynecology, Yong Yoo Lin School of Medicine, National University of Singapore, Singapore 117597, Singapore

**Keywords:** intrauterine resuscitation, fetal reserve index, electronic fetal monitoring, Pitocin, fetal distress

## Abstract

**Objective**: Intrauterine resuscitation (IR) may be employed during labor to reduce emergency deliveries with concerns for fetal wellbeing emanating mostly from increased uterine contraction frequency and/or intensity. However, there is no standard definition of what constitutes IR, and how its impact is assessed. Here, we have created two measures of relative IR effectiveness, determined over a two-hour time frame after Pitocin was first initiated, and asked how fetal risk severity at the time of its initiation impacted IR effectiveness and the clinical decisions made. **Methods**: We analyzed 118 patients receiving Pitocin who underwent IR at least once during labor. Retrospectively, we assessed risk levels using our Fetal Reserve Index version 2 (FRI v2) scores that were calculated in 20 min timeframes. FRIv2 scores include various maternal, obstetric, and fetal risk factors, uterine contraction frequency, and FHR baseline rate, variability, accelerations, and decelerations. We define 3 IR scenarios to assess relative IR effectiveness. (1) No reduction in PIT infusion rates (PITSAME), (2) decreased PIT infusion rates (DPIT), or (3) PIT turned off (PIT OFF). Maternal repositioning and oxygen administration are nearly universal across all types and, therefore, are not considered in groupings. We then created two measures of IR effectiveness by classifying changes in FRI v2 scores over six 20 min windows coincident with and following IR use as (1) “Improvement” (improvement relative to the FRIv2 score at IR initiation) and (2) “Stabilization” (no further decrease in FRI score relative to the FRIv2 score in the sixth 20 min epoch after IR initiation). We evaluated the relative effectiveness of the three PIT options, and to test whether the level of fetal risk at the time of IR initiation affected its short-term effectiveness, FRI v2 risk scores were assigned to one of three groups (Green [1.00–0.625]; Yellow [0.50–0.25]; Red 0.25–0.0]). Higher scores indicate lower risk. Statistical analysis was performed with ANOVA and t- tests. **Results**: Overall, the first and/or the only initiation of IR resulted in improvement in 71% of cases and stabilization in 78% of cases. The remaining 22% were failures, meaning that the FRIv2 score in the 6th 20 min period was lower than the score at the time of initiation. There were modest, but not statistically significant, differences in effectiveness (improvement or stabilization) by type of IR. There was a trend toward lower IR effectiveness of PIT OFF during IR initiation when compared to PIT continuation or decreased groups. **Conclusions**: IR initiation or type did not vary significantly by retrospectively calculated levels of fetal risk, showing that wide variation in clinician practices, not necessarily correlated with what we believe actual risk was, determine how IR was used. The FRI provides contextualization of FHR elements by adding maternal, fetal, and obstetric risk factors, and increased uterine activity enables a more rigorous and reproducible approach to analysis of emerging fetal compromise and IR effectiveness. As practice has shifted from the over-aggressiveness of PIT use to now premature discontinuations with any tracing variation, we need better metrics. FRIv2 further improves its physiologic underpinnings. Thus, we propose a new approach to the overall assessment of IR practice.

## 1. Introduction

Over the past four decades, Pitocin (PIT) has been increasingly used to induce or augment labor [1]. Consequently, the incidences of increased uterine activity (IUA) and its adverse sequelae have also increased. Considerable variation exists across and within labor and delivery units in the use and management of PIT infusions, with potentially counterproductive or even harmful protocols, such as turning off PIT when cervical dilatation has reached 5–6 cm or fetal risk is adjudged to be in the ACOG Category System’s CAT II range [2,3]. Such protocols are incompatible with the use of advancing technology to measure and assess the extent to which labor progress and fetal/maternal risk are in balance. Furthermore, no uniform standards exist for the quantitative assessment of intrauterine resuscitation (IR) components or for evaluating its efficacy, although some attempts are being made [2]. Therefore, it is virtually impossible to draw generalizable conclusions about its effects. Clearly, the increased use of PIT has directly generated more cases of increased uterine contractions and clinical hyperstimulation, partly contributing to the increase in emergency cesarean and vaginal deliveries [3]. The management of such cases involving PIT has resulted in increased use of IR with no uniformity of performance or evaluation.

IR is mostly initiated to reduce the risk of fetal hypoxia associated with increasing contraction frequency and intensity from PIT [4]. Besides the lack of IR standardization, assessing its impact is also complicated by the use of multiple components of interventions in differing combinations, e.g., stopping or reducing PIT infusions, shifting maternal position, administering oxygen, and, occasionally, tocolytic agents or amnio-infusions. Furthermore, decisions to continue, reduce, or stop PIT as part of IR are often made without even moderate evidence to justify differing approaches.

There are at least two major weaknesses in contemporary IR usage. First, most IR studies consider such interventions as a response to “fetal distress”, another term lacking a uniform definition, although it is often associated with FHR changes warranting at least an ACOG Category II (CAT II) classification. CAT II encompasses the majority of all pregnancies, rendering it a very poor statistic for discrimination of fetal status [5,6,7,8,9,10]. Second, the benefits and potential risks of several of the IR components, particularly maternal oxygen administration, have been seriously questioned and are hard to quantitate [8,10,11,12]. Ideally, an equilibrium should be established between PIT utilization and the need for IR to allow labor to progress safely [13]. Efforts to study systematically such a balance have focused on longer-term effects of different combinations of IR and PIT usage. These do not provide an assessment of the short-term effects of various clinical management options, which are required for evidence-based modulations of real-time patient care.

This study focuses directly on the measurement of the short-term effectiveness of different IR/PIT combinations as a way of focusing attention on one of the major elements of IR, the use of PIT. We evaluated differing methods of IR in the short term, defined here as a two-hour time period divided into six consecutive 20 min windows beginning with IR initiation.

To quantify fetal risk, we used the Fetal Reserve Index version 2.0 (FRIv2, described later) to address two questions regarding the short-term effectiveness of differing IR approaches:

1. In the short term, are continuing, reducing, or stopping PIT equally effective in halting the predictable downward trajectory of FRI risk scores and acid-base balance often associated with labor [9,14]?
2. How do IR short-term success rates compare following its initiation during periods of different risk levels as determined by FRIv2: low risk [green], moderate risk [yellow], or high-risk [red]?

## 2. Methods

We retrospectively studied 118 cases from our clinical database, collected over years at both university and community hospitals, involving term singleton patients. These cases were used for quality control and then academically studied with de-identified data and under IRB approval/exemption. PIT was used to induce or augment labor, and IR was used at least once (Table 1). Of these cases, 64 (54%) had more than one round of IR; the mean IR was 1.65; and IR administration episodes ranged from 1 to 6 separate occurrences. Here, we focused only on the initial IR usage, as the numbers of repeated IR rounds in each group were too small to compare with those receiving at least one IR round. In addition, the initial IR round was followed for two hours and deemed long enough to determine at least its short-term impact on fetal condition. FRIv2 scores (explained below) were retrospectively calculated beginning at the time of initiation of the first IR use and then repeated over six subsequent 20 min windows.

**Figure 1:**
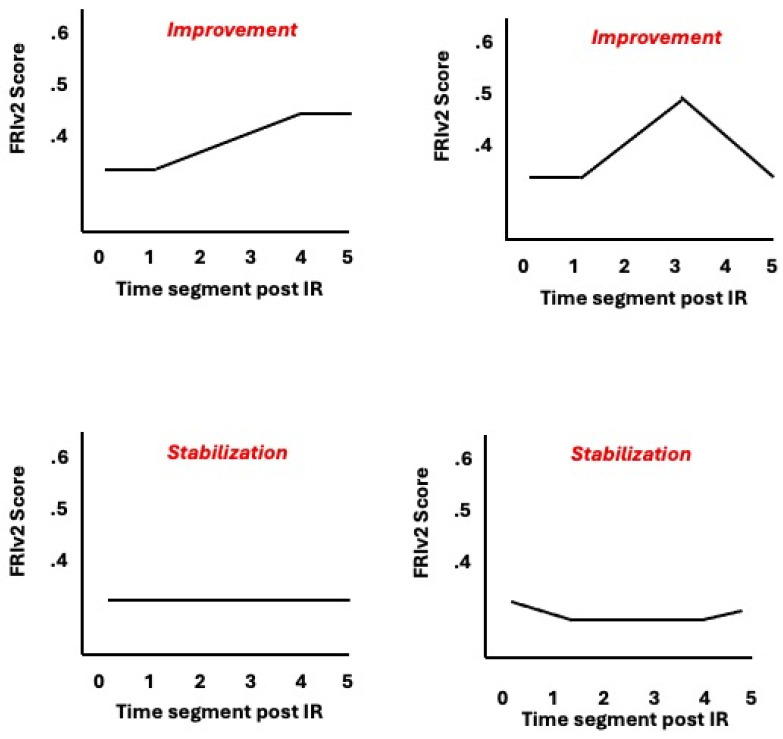
Graphs of post IR1 examples showing Improvement and Stabilization codes, where t_0_ = segment in which IR is initiated and t_5_ = 5th segment after initiation.

We defined two patterns of short-term success for IR:

1. *Improvement*: at least a temporary improvement in FRIv2 score without a worsening of risk relative to onset of IR initiation over the six subsequent windows.
2. *Stabilization:* no worsening in the level of risk relative to its level at IR initiation at the end of the sixth 20 min window. Stabilization is a more inclusive category for success because it includes all cases of Improvement and, also, all other cases in which the risk level did not worsen by the end of the sixth window. Examples of IR response curves are shown in Figure 1.

Multiple close measurements (timeframes) require more than just basic statistical analysis. Our approach for the statistical evaluation of IR impact on FRI scores over short time intervals was guided by Mathews et al. [15] They studied error dependence among scores in series measured at such short, sequential time intervals. Their work suggested several “summary statistic” methods for decoupling closely measured scores to resolve the following problem: calculating a mean, recording the highest score, measuring the amount of time needed to get to the maximum score, and determining the area under the curve. We retained the general principles of these summary statistics but eliminated some of their other suggestions because of two problems that show up when the starting point for scores varies. The first is the bias introduced by the level of the initial score—the higher the starting score, the greater the chance of a relatively high concluding score, even if IR usage had no effect whatsoever. The second is the problem of division by zero for scores that are constant. The starting-level bias problem can be eliminated through standardization, but the division-by-zero problem eliminated the area under the curve as a possible metric since too many cases are lost due to constant scores needing division by zero. To resolve this issue, we summarized the shape of the scoring curves over the two hours following the IR usage. This approach has two advantages: (1) it decouples the dependency among closely measured scores; (2) it creates the possibility of displaying such curves in real time to help clarify the impact of various clinical interventions.

For each case, we noted the specific IR approach options while PIT was being administered: (1) continuing Pitocin at the same infusion rate (PIT-CON); (2) reducing Pitocin infusion rates (PIT-D); (3) turning off Pitocin infusions (PIT-OFF). All options were accompanied by changing maternal position and administering supplemental oxygen, so these latter IR components were not considered as variables. We also did not score cases involving amnioinfusion, whether combined with either PIT-D or PIT-OFF, since there too few cases (<10%) to make meaningful comparisons. We also determined, using FRIv2, the levels of maternal, fetal, and obstetric risks at the time of IR initiation.

Our previous publications have used FRI version 1.0 (FRIv1), which contextualized FHR data by including information on risk factors (maternal, fetal and obstetric) and increased intrauterine activity or IUA (defined as more than four contractions per 10 min) [9]. The resulting summary scores varied from 0 to 1.0 and allowed measurements of fetal risk over the course of labor. In FRIv1, all risk factors received equal weight, which we knew was a convenient starting point for hand-calculated scores that would eventually be improved by weighting the variables. Even with that limitation, FRI recognition of fetal risk was considerably improved over the Category system [16].

In FRIv2, IR, EPI (epidural analgesia), and AROM were considered interventions rather than risk factors, which disentangles the interrelationships between risks and interventions. Also, rather than counting only the first-noted risk factor in each domain [maternal, fetal and obstetric], all risk factors were counted. Each identified risk factor at detection subtracts 0.125 from an initial starting point of 1.0 (indicating no apparent risk). The result of such calculations means that there is considerable variation in risk scores, even at admission. For our sample, the mean FRIv2 risk score at admission was 0.761 (SD +/− 0.141), and ranged from 0.375 to 1.0. Abnormal FHR features and IUA associated with PIT were more dynamic than other risk factors and received a 0.125 reduction for the first hour. If the dynamic risk factors lasted for more than one hour, they had another deduction of 0.125, so that 0.250 was included for subsequent, sequential appearances, thus giving more weight to the abnormality. To assess the influence of risk level at the first IR use, we divided the FRIv2 scores into our three groups at that time: Green (1.0–0.625), Yellow (0.500–0.26) and Red (≤0.250).

One-way ANOVA and Correlations (STATA v.18) were used to analyze the data, with post-test individual comparisons (Bonferroni method) used when there were more than two categories of the factors being investigated. The STATA graphics program generated the graphs in Figure 1. This was a secondary analysis of cases from multiple centers collected for quality control, de-identified for analysis, and as such, the study qualified for exemption as conferred by the Biomedical Research Association of NY IRB. (#16-12-180-429).

## 3. Results

*IR Type and Success Metrics*: By inference, when providers appeared to have only moderate concerns about fetal tolerance to PIT, and PIT infusion rates were more likely to either be maintained or only lowered rather than discontinued. These two IR approaches tended to be used earlier in labor. Most of the cases in our series fall within mild to moderate levels of concern, as none were classified by the ACOG CTG Category system as CAT III. In total, 75% were CAT II, and 25% were CAT I, which are rates that are consistent with multiple studies using the Category system and our own experiences with the FRI.

In our cases, IR episodes occurred from one to six times. IR was used once in 20 cases when PIT (PIT-CON) continued until birth. PIT-D was used once in 30 cases. The average number of episodes was 1.65 overall, suggesting that PIT-OFF was rarely employed in IR as the first instance of IR use. This result is consistent in several randomized controlled trials [17,18,19,20]. Our time frame for analysis was 2 h after PIT initiation. Any changes in PIT occurred only after 2 h.

As fetal risk levels increase, the likelihood of using a reduced PIT infusion as an IR component also increased. Because our clinical case dataset did not have meticulous recording of all cervical dilatation measurements, our working assumptions about stage of labor were inferred by a comparison of FRIv2 risk scores from the beginning of labor until the first IR usage. For PIT-OFF, the timing of the first use of IR was significantly later into the labor tracing (17.84 h, F = 6.06, *p* < 0.003) than for either IR with no PIT reduction (9.46 h) or for IR with PIT-D (11.66 h). A Bonferroni check on the pairwise differences confirmed this assertion.

We then examined the relationships between IR type and success metrics (Table 2). As expected, the PIT-CON and PIT-D cases had very similar FRIv2 scores at the time of implementation. Both had a mean FRI of 0.45, putting them close to the middle of the yellow zone when IR was started. Both groups had success rates of at least 75% by achieving either improved or stabilized status.

Pit-CON during IR was associated (not surprisingly) with significantly higher FRIv2 scores (lower risk) than with PIT-OFF, which had a mean of FRI score of 0.260 at IR initiation. This level is just above the red zone, which begins at an FRI score of 0.250. Even for those cases with the greatest risk at the first initiation of IR, improvement occurred 58% of the time in the five 20 min windows after the start of IR. The stabilization rate was 67%.

Since the risk of hypoxia increases considerably in the second stage of labor, had an expected risk reduction during the second stage been used as a counterfactual, the results for PIT-OFF would have been even more positive [14]. When the risk level was high (n = 32), there were 10 cases in which PIT was continued unchanged, 15 cases of PIT-D, and 7 cases of PIT-OFF. At the first IR of the 12 cases of PIT-OFF (about 10% of all cases), seven were red zone, four were yellow zone, and one was green zone. Continuing administration of PIT until delivery constituted 23 (19.5%) of the 118 total cases using IR.

*Influence of Risk Level at Time of IR Intervention*: We next compared success rates when IR began as a function of the level of risk, which varied from low [green] to moderate [yellow], to high [red]. There were no differences in the percentage of improvement or stabilization of FRI scores based on the level of risk at the time of IR initiation. (Table 3) For low-risk cases, IR achieved 76% improvement and 89% stabilization. For moderate-risk cases, the comparable figures were 67% and 75%, and for high-risk cases, both were 79%.

## 4. Discussion

Fifty years ago, Mondalou et al. [14] demonstrated that during labor, fetal acidosis, as measured by scalp pH and base excess, increased as fetal risk levels, by methods available at the time, appeared to rise to the clinicians managing the pregnancies. We found the same results in our studies, whose data partially overlapped those of Mondalou’s [21,22,23]. The extent of fetal deterioration and its slope downward become steeper in the second stage of labor. Notably, risk levels continue to increase even after delivery for the neonate for at least four minutes before starting to decrease. We have previously replicated these findings using FRI metrics with the gradual worsening of FRI scores before birth, which continues postnatally for four to eight minutes [9].

Analogous to cruise control in automobiles, variations in PIT infusion rates are intended to maintain the necessary equilibrium between the rate of labor progress and the avoidance of PIT-caused deleterious side effects, such as fetal hypoxia and acidosis. Our data show that fetal risk levels appeared to have little or no impact on the initiation or short-term effectiveness of the first use of IR. Thus, IR with varied PIT usage can be effective across various fetal risk levels. A second and more important conclusion is that IR can be a useful intervention for preventing fetuses from reaching higher-risk status and for lifting them out of high-risk status, or at least lowering their presumptive risk when they enter the high-risk red zone (FRIv2 score range from 0.0 to 0.250).

Once PIT has been started, PIT management options during IR varied considerably, possibly reflecting the providers’ a priori assessments of intrapartum fetal status. Our dataset revealed multiple uses of various forms of IR that had poor correlation with our retrospective assessment of risk levels by FRIv2 scores, demonstrating vast differences in how PIT was managed in actual practice.

The ACOG Category system was developed to improve neonatal outcomes by standardizing what constitutes a concerning fetal heart rate pattern allowing for earlier recognition of concerns by all personnel (nurses, midwives and obstetricians). Many authors, including us, have criticized the Category system as a poor screening test, [9,21,22,23]. Modern clinical obstetrical intrapartum care particularly struggles to manage the Category II tracings, in particular. In many labor and delivery units (community and university), both nurses and doctors often disagree on the interpretation of Category II FHR tracings. The well-intentioned algorithm from the California Maternal Quality Care Collaborative (CMQCC) [24] has, inadvertently, further complicated the management of labor. In actual clinical practice, there is, too commonly, an inappropriate interruption and discontinuation of Pitocin augmentation triggered by lesser experienced staff in the spirit of teamwork and enhanced safety. The net effect is paradoxically an increase in unnecessary cesarean deliveries. Clearly, additional tools are needed to aid practitioners in the safe conduct of labor. The FRI, especially in further refined and validated iterations, provides a real-time functional tool to better determine the fetal status, trajectory of fetal reserve depletion, recovery of reserve, following resuscitative measures [5].

Our study articulates a serious need to improve assessment of perceived risks in labor. As multiple undefined, arbitrary, unstandardized, and often undocumented methods for risk determination were employed at IR initiation, risk-related assessment of IR effectiveness is difficult at best. This underscores the need to develop standardized methods for risk assessment and subsequent management. Comprehensive fetal risk assessment methods, such as the FRIv2 scoring system, have the potential to refine risk assessment and could serve in the future as an objective platform to objectively evaluate IR effectiveness. At the very least, we hope to establish a “common language” around which comparisons can be made.

Our study’s findings are based on a limited number of cases for which retrospective contextualization of FHR data was performed manually. With future data computerization and using AI enhancements, providers will be able to watch risk curves unfold over time and could provide earlier, more appropriate and nuanced interventions. Therefore, newer management approaches will depend on the development of an adaptive system, whether within a sociotechnical system framework [25] or a learning health system framework [26]. This could lead to a platform that can integrate risk information from both electronic and non-electronic sources and be displayed in a manner that leads to greater acceptance, use, understanding, and effectiveness over time [25].

Given these findings, we propose an FRIv2-based scoring system approach to gauge its effectiveness in future studies. This would be based on establishing FRI risk scores prior to IR initiation and, once IR is begun, using a time-based, stepwise trajectory of the successive scores. Such a prospective approach might resemble the following points:

1. FRI Green Zone (score 1.0–0.625). Continued observation but withholding IR unless the fetus enters the Yellow Zone.
2. FRI Yellow Zone (score 0.50–0.26). Initiate IR and compare subsequent scores in consecutive 20 min windows for evidence of improvement or stabilization. Discontinue IR if fetus returns to the Green Zone, or continue IR if there is stabilization.
3. FRI Red Zone (0.25–0.0). Initiate IR with 20 min window comparisons of score trajectory. Continue IR if fetus returns to Yellow Zone. Move to delivery if the fetus does not return to Yellow Zone within 40 min or if the score continues to decrease.

We also suggest that future clinically oriented research on this topic might consider a standardized approach, including a three-dimensional typology with axes representing the level of risk at IR use (Green, Yellow, Red, for example), the rate at which the risk of hypoxia is progressing (perhaps more simply, the first or second stage of labor), and the concomitant management of PIT infusions, i.e., PIT-CON, PIT-D, or PIT-OFF.

## 5. Strengths and Limitations

The three main strengths of this study are (1) the development of metrics for gauging the impact of IR on the level of risk/FRI score, (2) the assessment of the short-term impact of IR in the context of consequent PIT management, and (3) the establishment of a conceptualization framework for studying the relationships among level of risk and responsiveness to treatments.

Limitations include the inability to specify from our data all factors relevant to an assessment of IR due to a lack of precise information on the initial rates of PIT infusion or their rate reductions when they occurred. Also, we cannot determine maternal hypotension, which often results from epidurals, nor IV flow rates and volumes. Furthermore, our sample lacked consistent measures of labor progress with respect to cervical dilatation, except for change in labor stage, and there was only anecdotal evidence that maternal repositioning and oxygen supplementation were performed. The first limitation only suggests that while in selected cases, alteration of PIT administration could play an adjunctive role in IR, larger, more precise studies will be needed to clarify this point. The second and third limitations can all be addressed by the FRI system with the IR metrics we propose [9].

## 6. Conclusions

In practice, IR use and evaluation in contemporary practice vary considerably, making it difficult to properly assess its utility. To make IR use better focused and more standardized, quantitative fetal risk levels should be considered before instituting this intervention.

At all risk levels, IR appears to exert a positive effect on the stabilization or actual improvement of fetal condition. To make IR use better focused and more standardized and precise, quantitative fetal risk levels should be considered before instituting this intervention [9,21,22,23]. This will also make adjustments in PIT usage more rational for patients receiving the drug. Adapting our FRIv2 scoring system to improve the use of IR will be a goal of future studies and will, hopefully, generate enough data to enable better practice guidelines.

## Figures and Tables

**Table 1:** Categorizations and Results of IR (*n* = 118).

Variable	Mean	Percent
	(SD)	
IR type		
Continued level of Pitocin (PIT-CON)		33%
Reduction in Pitocin (PIT-D)		56%
Stopping of Pitocin (PIT-OFF)		11%
FRI grouping at start of IR		
Green (0.625–1.0)		24%
Yellow (0.375–0.500)		46%
Red (0.000–0.250)		29%
Improvement		
No improvement		28%
Improvement		72%
Stabilization		
No stabilization		21%
Stabilization		79%
Total red zone minutes	137.97	
	(238.43)	

**Table 2:** Success Metrics by IR Associated with PIT Administration Options.

	Percentage Improved *	Percentage Stabilized **
	(SD)	(SD)
(PIT-CON) PIT Continuation (*n* = 39)	76%	78%
	(44)	(42)
(PIT-D) PIT Reduction (*n* = 64)	75%	84%
	(44)	(37)
(PIT-OFF) Pit Off (*n* = 12)	58%	67%
	(52)	(49)

* F = 0.78, *p* < 0.46; ** F = 1.10, *p* < 0.34.

**Table 3:** Success Metrics by FRIv2 Risk Level ^#^ at Labor Duration Point of IR Administration.

	Percent Improved *	Percent Stabilized **
	(SD)	(SD)
Green (*n* = 29)	76%	89%
	(44)	(31)
Yellow (*n* = 55)	67%	75%
	(47)	(44)
Red (*n* = 34)	79%	79%
	(41)	(41)

^#^ The FRI varies from Green (low risk, 0.625–1.000), through Yellow (moderate risk, 0.375–0.500), to Red (high risk, 0.000–0.250). * F = 0.86, *p* < 0.43; ** F = 1.33, *p* < 0.27.

## Data Availability

We are in the middle of several studies with these data which will be made available when all are done.
